# Bilateral open lip schizencephaly

**DOI:** 10.1016/j.amsu.2021.103204

**Published:** 2021-12-23

**Authors:** Turyalai Hakimi, Khalid Mohammad Qasem

**Affiliations:** a Department of Pediatric Surgery, Kabul University of Medical Science, Maiwand Teaching Hospital, Kabul, Afghanistan; b Department of Orthopedic Surgery, Kabul University of Medical Science, Ali Abad Teaching Hospital, Kabul, Afghanistan

**Keywords:** Schizencephaly, Developmental disorder, Cleft, Closed lip, Open lip, Ventriculomegaly

## Abstract

Schizencephaly is a central nervous system (CNS) developmental disorder characterized by abnormal cleft extending from the lateral ventricles to the cerebral cortex. Clinically, it occurs as *trans*-mantle, closed lip and open lip types which may be unilateral or bilateral. The exact cause of schizencephaly is not known but genetic disorders, exposure to teratogens, viral infections and maternal age are implicated. We present a case of bilateral open lip schizencephaly with some degrees of neurological disorders caused by increased intra-cranial pressure (ICP) due to ventriculomegaly. We applied ventriculo-peritoneal shunt (V–P shunt) to the patient with considerable improvement after post-operative follow-up.

## Highlights

• " Schizencephaly " is a rare problem in children with variety of neurological and motor deficits.
• Schizencephaly is mostly associated with seizures but it could be existed without seizures.
• Mental and physical rehabilitation is the main goals of treatment but in elevated intra-cranial pressure, intra-cranial shunting is advisable.
• In developing nations, viral infections, addiction to narcotics and familial violence as trauma could be implicated.

## 1 Introduction and importance

Schizencephaly is a rare congenital anomaly characterized by the failure of neuronal migration and resultant cleft extending from cerebral hemispheres to neuroepithelial cells lining the lateral ventricles [1,2]. The prevalence of schizencephaly is 1.48/100 000 live births which is more common in younger mothers [3]. The etiology is not well understood but teratogens, viruses mostly cytomegalovirus, alcohol and narcotics, hypoxia, maternal age and trauma may be involved [[4], [5], [6]]. Most of the schizencephaly cases are sporadic and non-familial with no any known causative etiology [14]. Computerized tomography scan and Magnetic resonance imaging (CT-Scan and MRI) are the modalities of choice for diagnosis but MRI is superior for better differentiation between gray and white matters [7,8]. Treatment is supportive and surgery is needed if there is hydrocephalus [7].

## 2 Case presentation

**Fig. 1:**
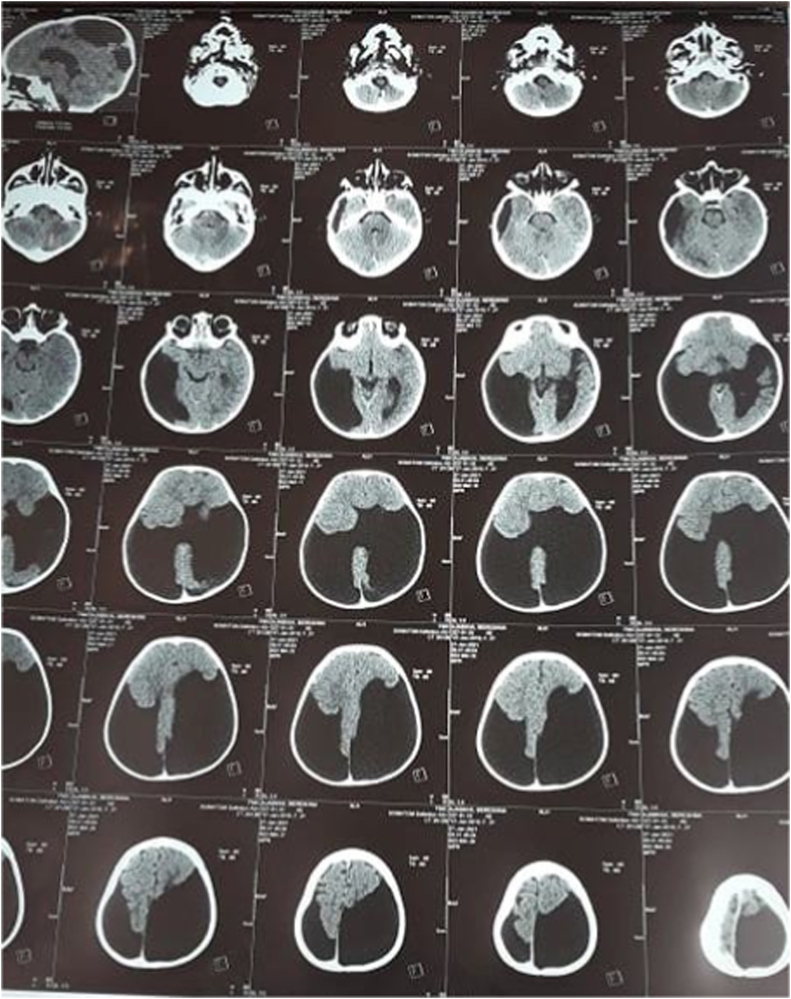
CT-Scan image, showing cleft extending from Pia mater to the lateral ventricles.

**Fig. 2:**
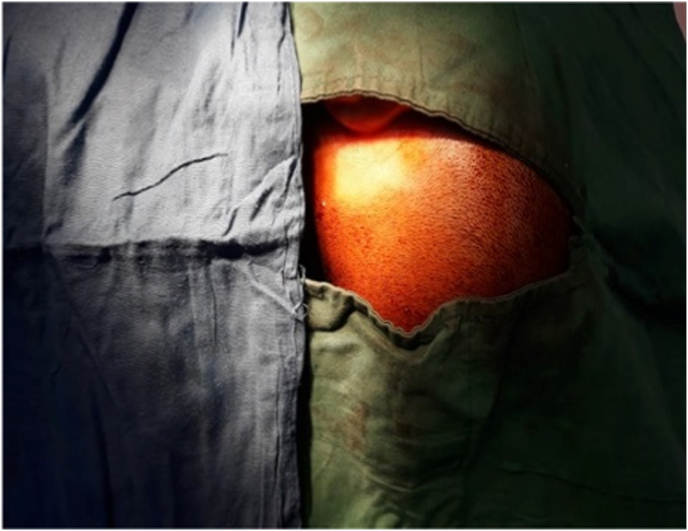
Prepared right temporo-Parietal area for incision.

**Fig. 3:**
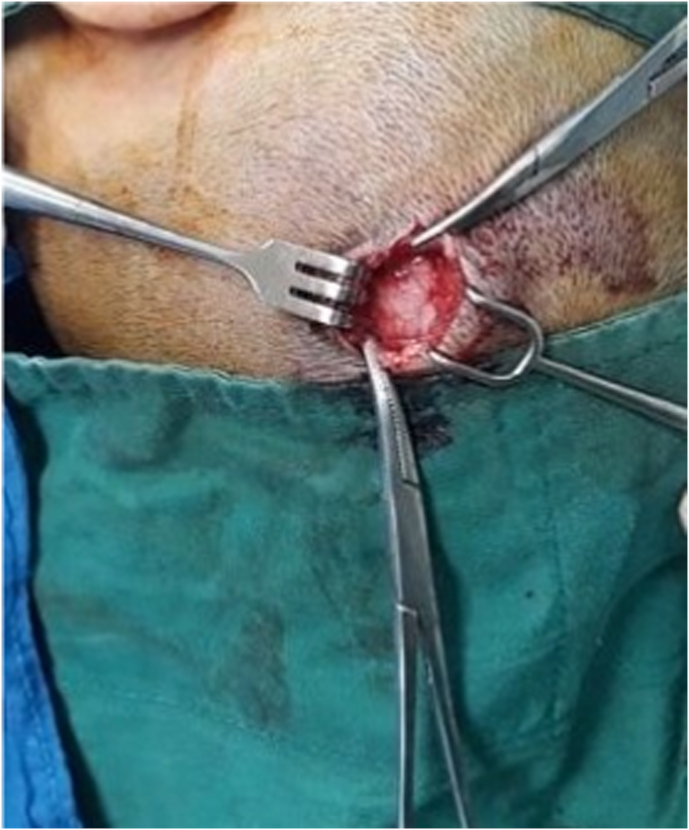
Scalp incision.

**Fig. 4:**
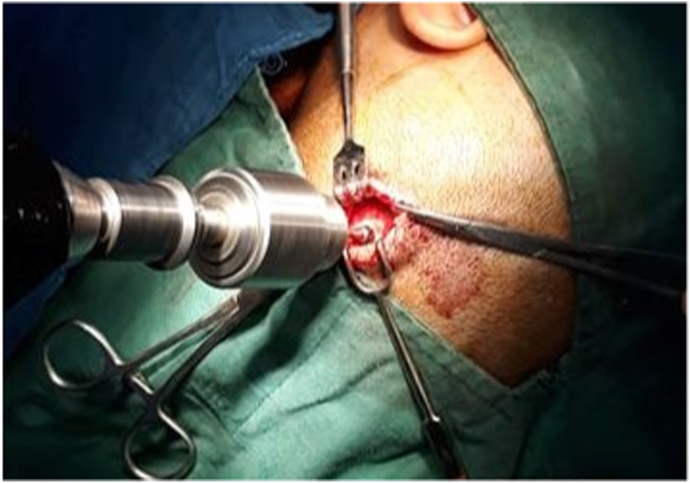
Skull bur hole using drill.

**Fig. 5:**
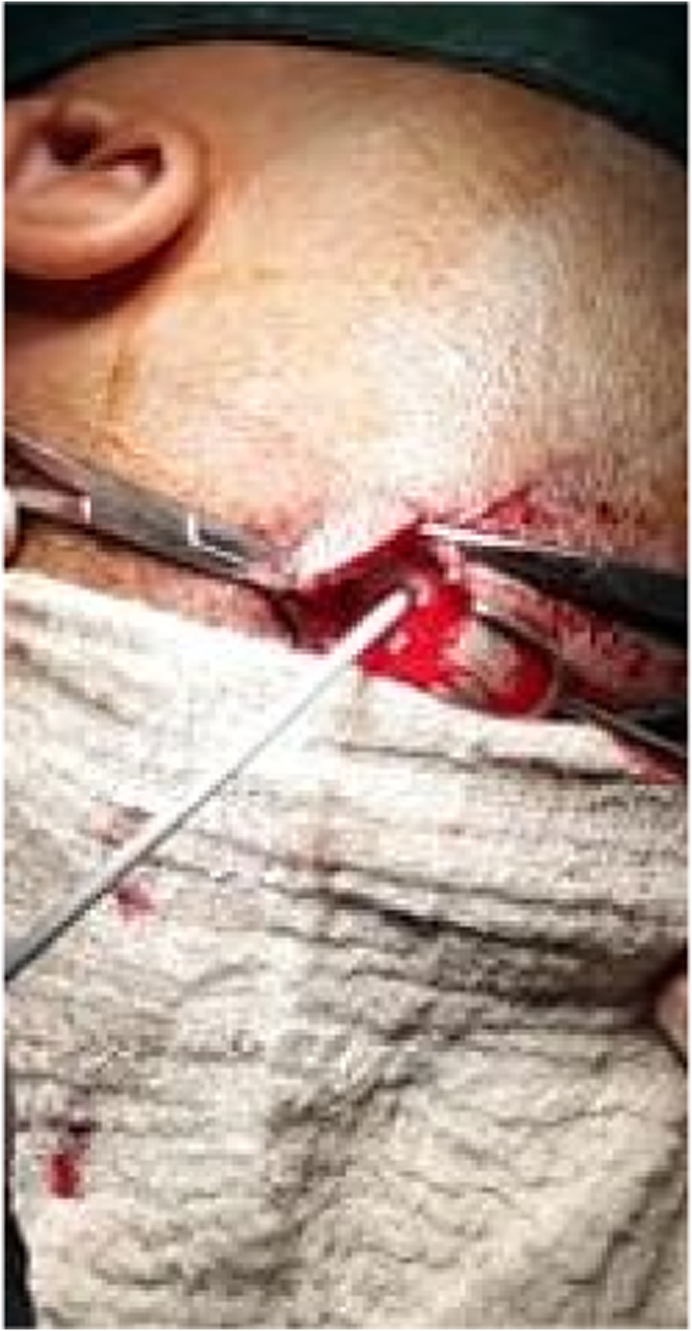
V–P Shunt application (insertion process of ventricular end of shunt along with guider).

**Fig. 6:**
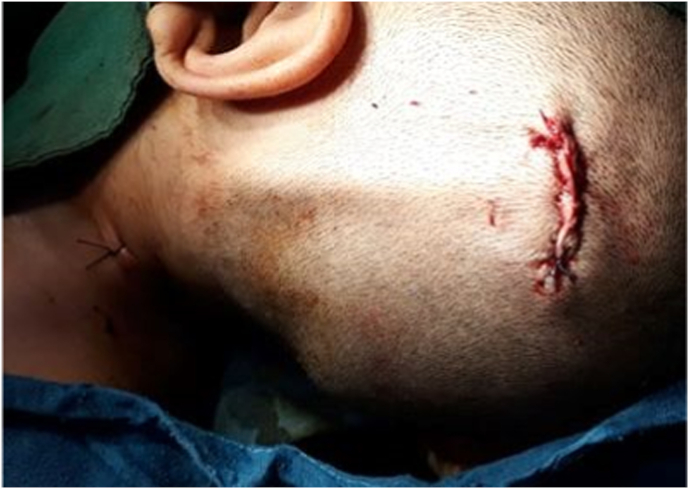
Wound closure in the neck and head.

We present a case of 30 months old child brought by her parents to our department suffering from head enlargement associated with internal strabismus and lower limbs low degree weakness. The patient was born to a consanguineous couple with no familial history for the mentioned problem. According to the patient parents information, the child head started to expand with the eye balls moving toward midline at the age of 4 months. They took their child to the local clinic and received appropriate advice by the relevant physician. In the next visit for the purpose of definite treatment, the patient was referred to another physician who had private practice in the downtown. Here the patient was taken under regular conservative treatment with fixed intervals. This process lasted for two years with no positive result but worsening the condition, so the patient was referred for specialized treatment to our hospital pediatric surgery department. Our team evaluated the patient by performing the relevant clinical examinations. Patient was weighting 11kg and with physical exam her head was enlarged with the internal strabismus and lower limbs weakness but all reflexes still remained intact and no history of seizures (common in schizencephaly). Our team carried out all biochemical tests along with brain CT-Scan. Biochemical tests were normal, but CT-Scan showed bilateral cysts separating by a cleft, resembling bilateral open lip schizencephaly (Fig. 1). As there was considerable ventriculomegaly, our team advised VP-Shunt for the purpose of decreasing ICP. We treated the patient by placement of VP-Shunt with no any intra-operative and post-operative complications (Fig. 2, Fig. 3, Fig. 4, Fig. 5, Fig. 6). The operation ended uneventfully.

## 3 Discussion

Schizencephaly is uncommon cortical anomaly resulting from slit brain due to the cleft extending from pia matter to the ependymal cell of lateral ventricles. The condition was first described by Yakovlev and Wadsworth in 1946 as the infolding of gray matter along the clefts which are located in the area of primary fissure and associated with other CNS anomalies [15]. Closed lip type of schizencephaly is characterized by inter-connected gray mater lined lips whereas the open lip type is characterized by open lips with a CSF filled cleft extending to the lateral ventricles [9]. Our case was bilateral open lip type of schizencephaly.

The cause is unknown but failure of neuronal migration along the radially oriented glial cells in the brain cortex due to any type of insult and resultant hypoxemia with infarction in the 8th week of gestation is hypnotized [9,10]. Additionally viral infection (cytomegalovirus) and more recently Zika virus, attempted abortion, hypoxia, maternal age and trauma are also involved [6,11,16].

Signs and symptoms depend on the clinical type. Closed lip schizencephaly is manifested by hemiparesis and motor delay whereas open lip schizencephaly manifested by hydrocephalus and seizures [12]. Our patient was complaining of diplegia, internal strabismus and hydrocephalus but not history of seizures (rare).

Definite diagnosis is given by history, signs, symptoms and imaging modalities (CT-Scan & MRI). Treatment is mostly supportive focusing on seizures control and rehabilitation of physical and mental status. Surgery in not needed but if there are signs and symptoms of increased ICP especially hydrocephalus, V–P Shunt application is mandatory [13]. Conditions such as focal cortical dysplasia with cleft in the cortex but not extending to the ventricles, gray matter heterotropia seen as a linear cleft but periventricular gray matter generally bulge into the ventricles and procencephaly extension from the cortex to ventricles but lined by gliotic white matter are all that should be considered in differential diagnosis of schizencephaly [17,18]. As our patient was suffering from hydrocephalus, we applied V–P Shunt and the operation ended uneventfully. Six months following discharge from the hospital, the patient's mentioned complains considerably relieved (patient's condition description by her father through phone contact).

## 4 Conclusion

Schizencephaly is still prevalent in developing nations and on time management will improve the patient condition and prevent disability. Our case was from a rurally lived illiterate family and low socioeconomic condition with no direct access to primary healthcare system and referral to specialized center. We assume that antenatal viral infections (ante-natal viral screening is not done routinely in rural areas), addiction (not alcohol but narcotics which are common in some families where they cultivate opium in their farms and become addicted), maternal age and sometimes (maternal trauma due to violence) may be implicated as causes. Further research should be carried out to know more about the causes of CNS anomalies in developing nations.

## Ethical approval

No ethical approval was necessary.

## Sources of funding

No fund and grant.

## Author contribution

Turyalai Hakimi (TH) performed the procedure and conceptualized the manuscript. TH and Khalid M, Qasem (KMQ) designed the study. TH and KMQ contributed for the diagnosis and treatment. Both TH and KMQ wrote the original draft and edited the manuscript with the supervision of the entire study process. Both authors read and approved the final manuscript.

## Registration of research studies

1. Name of the registry: None.

2. Unique Identifying number or registration ID: None.

3. Hyperlink to your specific registration (must be publicly accessible and will be checked): None.

## Guarantor

Dr. Turyalai Hakimi.

## Consent

Written informed consent was obtained from the patient for publication of this case report and accompanying images. A copy of the written consent is available for review by the Editor-in-Chief of this journal on request.

## Source of funding

None.

## Authorship

All authors attest that they meet the current ICMJE criteria for authorship.

## Declaration of competing interest

The authors declare that they have no known competing financial interests of personal relationships that could have appeared to influence the work reported in this paper.
